# Increased brain gyrification and subsequent relapse in patients with first-episode schizophrenia

**DOI:** 10.3389/fpsyt.2022.937605

**Published:** 2022-08-10

**Authors:** Daiki Sasabayashi, Yoichiro Takayanagi, Tsutomu Takahashi, Atsushi Furuichi, Haruko Kobayashi, Kyo Noguchi, Michio Suzuki

**Affiliations:** ^1^Department of Neuropsychiatry, University of Toyama Graduate School of Medicine and Pharmaceutical Sciences, Toyama, Japan; ^2^Research Center for Idling Brain Science, University of Toyama, Toyama, Japan; ^3^Arisawabashi Hospital, Toyama, Japan; ^4^Department of Radiology, University of Toyama Graduate School of Medicine and Pharmaceutical Sciences, Toyama, Japan

**Keywords:** gyrification, local gyrification index, relapse, first-episode schizophrenia, prognostic biomarker, magnetic resonance imaging

## Abstract

Most schizophrenia patients experience psychotic relapses, which may compromise long-term outcome. However, it is difficult to objectively assess the actual risk of relapse for each patient as the biological changes underlying relapse remain unknown. The present study used magnetic resonance imaging (MRI) to investigate the relationship between brain gyrification pattern and subsequent relapse in patients with first-episode schizophrenia. The subjects consisted of 19 patients with and 33 patients without relapse during a 3-year clinical follow-up after baseline MRI scanning. Using FreeSurfer software, we compared the local gyrification index (LGI) between the relapsed and non-relapsed groups. In the relapsed group, we also explored the relationship among LGI and the number of relapses and time to first relapse after MRI scanning. Relapsed patients exhibited a significantly higher LGI in the bilateral parietal and left occipital areas than non-relapsed patients. In addition, the time to first relapse was negatively correlated with LGI in the right inferior temporal cortex. These findings suggest that increased LGI in the temporo-parieto-occipital regions in first-episode schizophrenia patients may be a potential prognostic biomarker that reflects relapse susceptibility in the early course of the illness.

## Introduction

Over 80% of patients with schizophrenia experience a relapse within 5 years of their first episode of psychosis (1) and the cumulative effect of relapse can result in exacerbation of psychotic symptoms, antipsychotic treatment resistance, and functional decline (2, 3). Effective relapse prevention contributes to better long-term prognosis of schizophrenia (4). Whereas maintenance treatment with antipsychotic drugs can significantly reduce relapse rates in patients with schizophrenia (5), there is a subgroup of schizophrenia patients who may only suffer a single psychotic episode and may not need maintenance antipsychotic medication (6). Evidence for antipsychotic treatment may drive the need to identify predictors of subsequent relapse in the course of schizophrenia (7). It has been reported that several clinical features, such as long duration of untreated psychosis (DUP), poor premorbid functioning, and severe negative symptoms, are associated with higher risk of relapse in schizophrenia (1, 8); however, objective biomarkers of relapse susceptibility have not been well documented.

Schizophrenia patients likely exhibit diverse brain structural changes even at their first episode (9), which may be associated with clinical features such as long-term outcome (10). As treatment response and relapse susceptibility may partially be defined by the initial period of schizophrenia (11, 12), through a neurodevelopmental pathology (13), brain morphological abnormalities closely related to early neurodevelopment (14) may partly be predictive of subsequent clinical course. As brain gyrification is mostly formed during fetal life and remains relatively stable after early childhood (15, 16), its anomalous patterns implicate pre- and perinatal neurodevelopmental insults (17–19). Previous magnetic resonance imaging (MRI) studies of brain gyrification have generally demonstrated increased gyrification index (GI) of widespread cortical areas in first-episode schizophrenia [reviewed by Matsuda and Ohi (20); Sasabayashi et al. (21)]. Furthermore, previous studies have revealed an association between altered gyral patterns in early psychosis (including the prodromal phase) and later psychosis onset (22, 23) or antipsychotic treatment response (24). Taken together, deviated gyral patterns observed in early stages of schizophrenia may be a potential biomarker for disease prognosis including subsequent relapses.

In the present study, we aimed to compare the gyrification pattern of the entire cortex between first-episode schizophrenia patients with and without relapse in the early course. We also explored the relationship among gyral characteristics and clinical variables including quantitative indicators for relapse. Based on previous findings in patients with schizophrenia that showed GI differences between treatment responders and non-responders (24), we predicted that relapsed and non-relapsed patients would differ in GI and that baseline GI changes in relapsed patients would be related to time to subsequent relapse.

## Materials and methods

### Study participants

Fifty-two patients with first-episode schizophrenia were recruited from inpatient and outpatient clinics of the Department of Neuropsychiatry at Toyama University Hospital. All participants were also included in our previous study that conducted group comparisons of the gyrification pattern between first-episode schizophrenia patients and healthy controls (25).

Based on a structured clinical interview by trained psychiatrists using the Comprehensive Assessment of Symptoms and History (26), each participant fulfilled the ICD-10 research criteria (27). Schizophrenia patients who experienced their illness onset within 1 year (*n* = 41) (28) or on their first psychiatric hospitalization (*n* = 11, duration of illness ≦ 41 months) were defined as first-episode patients. Their clinical symptoms were assessed using the Scale for the Assessment of Negative Symptoms (SANS) (29) and the Scale for the Assessment of Positive Symptoms (SAPS) (30) at the time of baseline scanning (*n* = 49) as well as follow-up (mean follow-up period of 2.7 years) (*n* = 14). At baseline, 50 of the 52 patients were receiving antipsychotics, one patient was antipsychotic-free at scanning but had a history of antipsychotic medication, and one patient was antipsychotic-naïve. Different typical and atypical antipsychotic dosages were converted into haloperidol equivalents using the guideline by Toru (31). Duration of untreated psychosis was defined as the duration from the illness onset to the initiation of antipsychotic treatment (32).

All participants were physically healthy and right-handed. Trained neuroradiologists screened their MRI scans for gross brain abnormalities. None of participants had a lifetime history of serious head injury, seizure, neurological disease, substance abuse, electroconvulsive therapy, or other medical conditions that influence mental condition such as steroid use, thyroid dysfunction, diabetes, or hypertension. The present study was approved by the Committee on Medical Ethics of the University of Toyama (No. I2013006). In accordance with the Declaration of Helsinki, written informed consent was obtained from all participants.

### Assessment of relapse

With reference to the criteria of Csernansky et al. (33), relapse was defined as a case of psychiatric hospitalization for worsening symptoms and/or for suicidal/homicidal ideation, deliberate self-injury, or violent behavior resulting in injury to another person or property damage that was judged clinically significant. All schizophrenia patients were clinically followed up for 3 years after MRI scanning and then divided into 19 patients who experienced relapse (Relapse) and 33 patients who remained relapse-free during the clinical follow-up (Non-relapse) based on clinical information from an electronic medical record system.

### Image acquisition and processing

All participants were scanned using a 1.5-T Magnetom Vision scanner (Siemens Medical System, Erlangen, Germany) with 3-D gradient-echo sequence fast low-angle shots (FLASH) yielding 160–180 contiguous T1-weighted sagittal slices of 1-mm thickness. Imaging parameters were as follows: repetition time = 24 ms; echo time = 5 ms; flip angle = 40°; field of view = 256 mm; matrix size = 256 × 256 pixels; and voxel size = 1.0 × 1.0 × 1.0 mm.

Using the FreeSurfer Software Suite (ver. 5.3.),^1^ the T1-weighted images were preprocessed by the FreeSurfer’s standard auto-reconstruction algorithm, which was involved in non-uniform intensity normalization, removal of non-brain tissue, affine registration to the Montreal Neurological Institute space, Talairach transformation, and segmentation of gray/white matter tissue (34). One trained researcher (D.S.), who was blind to the subjects’ identities, visually inspected each reconstructed image and manually corrected any segmentation errors.

The gyrification pattern of the entire cortex was indexed by the vertex-wise local gyrification index (LGI) value, which is a three-dimensional extension of classical two-dimensional GI measurement (35). Based on the method of Schaer et al. (36), we generated approximately 800 regions of interest (ROI; radius = 25 mm), which partly overlapped with each other and covered whole cortical areas, and computed the area ratio of the outer contour and the corresponding inner (pial) contour in each spherical ROI.

### Statistical analysis

Demographic differences between the two groups (Relapse vs. Non-relapse) were evaluated with a one-way analysis of variance or χ^2^-test.

We mapped each vertex-wise LGI value onto a common spherical coordinate system (fsaverage), which were smoothed with a 0-mm Gaussian kernel according to FreeSurfer Support recommendation.^2^ Using the Query Design Estimate Contrast application embedded in the FreeSurfer program, a general linear model controlling for age and sex was adapted to estimate the group differences of the LGI value at each vertex. Among the Relapse group, vertex-by-vertex LGI correlation analyses with relapse index, including numbers of relapse and duration between scanning and first relapse, were estimated using a general linear model controlling for age and sex. For both models, we also performed supplementary analyses that added medication dose and duration of medication as covariates. A Monte Carlo simulation embedded in the Analysis of Functional NeuroImages’ AlphaSim program (NIMH, Bethesda, MD, United States) was used to perform multiple comparisons (37). A total of 10,000 iterations of simulation were run for each comparison with a threshold of two-tailed *p* < 0.05 to define statistically significant clusters.

Furthermore, single regression analyses were performed to determine whether DUP was associated with altered gyral patterns especially in relapsed patients. Average LGI values in three clusters, where we found significant differences between Relapse and Non-Relapse groups, were used as the dependent variables, with DUP as the independent variable. The statistical significance level was set at 0.05 as well.

## Results

### Demographic background at baseline

The Relapse and Non-relapse groups did not differ significantly in age, sex, education, parental education, age at onset, duration of illness, and DUP. Total SAPS/SANS scores at the time of baseline scanning, daily dosage, and duration of antipsychotic medication were also comparable between the groups. The relapsed group tended to show higher Total SAPS/SANS scores at the time of follow-up than the Non-relapse group (Table 1).

**TABLE 1:** Demographic characteristics of study participants.

	Relapse	Non-relapse	Test statistics	*P*-value
	(*n* = 19)	(*n* = 33)		
Sex, male/female (*n*)	14/5	18/15	Chi-square = 1.87	0.172
Age (years)	23.7 ± 5.6	24.3 ± 4.4	*F*(1, 51) = 0.17	0.684
Height (cm)	166.2 ± 6.9	165.1 ± 8.0	*F*(1, 51) = 0.28	0.603
Education (years)	12.9 ± 1.9	13.9 ± 2.1	*F*(1, 51) = 3.30	0.075
Parental education (years)	12.8 ± 2.0	13.1 ± 2.1	*F*(1, 51) = 0.29	0.596
Age at onset (years)	22.8 ± 5.9	23.6 ± 4.4	*F*(1, 51) = 0.33	0.569
Duration of illness (months)	10.4 ± 11.6	9.1 ± 8.3	*F*(1, 51) = 0.20	0.658
Medication type (atypical/typical/mixed)	6/11/1 (*n* = 18)	9/23/0 (*n* = 32)	Chi-square = 2.08	0.354
Medication dose (haloperidol equivalent, mg/day)^a^	13.7 ± 11.8 (*n* = 18)	9.6 ± 6.9 (*n* = 32)	*F*(1, 49) = 2.49	0.121
Duration of medication (months)	8.6 ± 12.6 (*n* = 18)	8.4 ± 12.4 (*n* = 33)	*F*(1, 50) = 0.001	0.970
SAPS total at baseline scanning^b^	26.3 ± 21.5 (*n* = 18)	29.2 ± 23.8 (*n* = 32)	*F*(1, 49) = 0.19	0.663
SANS total at baseline scanning^b^	50.2 ± 23.2 (*n* = 18)	52.8 ± 27.3 (*n* = 32)	*F*(1, 49) = 0.12	0.729
SAPS total at follow-up^c^	31.6 ± 18.0 (*n* = 5)	15.7 ± 17.9 (*n* = 9)	*F*(1, 13) = 2.55	0.136
SANS total at follow-up^c^	52.4 ± 31.0 (*n* = 5)	27.1 ± 17.4 (*n* = 9)	*F*(1, 13) = 3.94	0.071
Numbers of relapse (*n*)^d^	1.1 ± 0.3			
Duration between scanning and first relapse (years)^d^	1.7 ± 0.7			
Duration of untreated psychosis (months)^e^	4.4 ± 5.2 (*n* = 18)	2.3 ± 3.1 (*n* = 33)	*F*(1, 50) = 3.08	0.086
Intracranial volume (ml)	1552.9 ± 155.4	1512.3 ± 144.7	*F*(1, 51) = 0.90	0.347^f^

Non-relapse, schizophrenia patients who remained relapse-free during the follow-up; Relapse, schizophrenia patients who experienced relapse during the follow-up; SANS, Scale for the Assessment of Negative Symptoms; SAPS, Scale for the Assessment of Positive Symptoms.

Values represent the mean ± SD unless otherwise stated.

^a^Different typical and atypical antipsychotic dosages were converted into haloperidol equivalents using the guideline by Toru (71).

^b^Data missing for two subjects.

^c^Data acquiring from fourteen subjects.

^d^Nineteen patients experienced relapses within 3 years after their first-episode of psychosis.

^e^Defined as the duration from the illness onset to the initiation of antipsychotic treatment (72). DUP was undetermined for one antipsychotic-naïve patient at the time of scanning.

^f^Age was used as a covariate.

### Group comparison of local gyrification index

**FIGURE 1:**
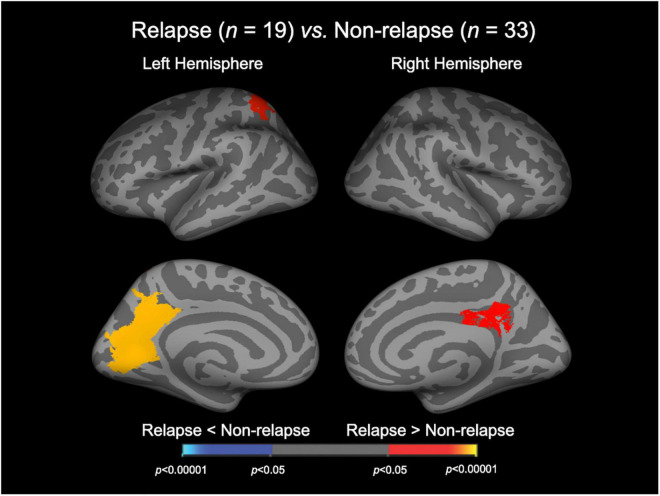
Cortical statistical maps displaying the comparison of local gyrification index between relapsed and non-relapsed patients with schizophrenia. The maps are shown for the right and left hemispheres in lateral (upper) and medial (bottom) views. Horizontal bar shows *p*-values (*p* < 0.05, corrected).

The Relapse group exhibited a significantly higher LGI in the bilateral precuneus cortices, bilateral isthmus cingulate gyri, left superior parietal cortex, left cuneus cortex, left pericalcarine cortex, and left lingual gyrus (Figure 1 and Table 2). There were no cortical areas where the Relapse group exhibited a significantly lower LGI than the Non-relapse group.

**TABLE 2:** Clusters with significant group differences in local gyrification index.

Cluster no.	Cluster size (mm_2_)	Cluster-wise *p (corrected)*	MNI coordinates			Annotation
			*x*	*y*	*z*	
**Relapse > Non-relapse**						
1	4022.36	0.0001	–14.1	–63.6	4.2	Left precuneus and cuneus cortex, isthmus cingulate gyrus, pericalcarine cortex, and lingual gyrus
2	707.23	0.0161	–18.8	–50.4	57.3	Left superior parietal lobule
3	646.85	0.0328	4.6	–40.6	27.4	Right precuneus cortex, posterior and isthmus cingulate gyrus

Non-relapse, schizophrenia patients who remained relapse-free during the follow-up; Relapse, schizophrenia patients who experienced relapse during the follow-up.

When we added medication dose and duration of medication as covariates, significant clusters were detected in broader regions than in the original analyses for LGI group comparison (Supplementary Figure 1).

### Relationship among local gyrification index and clinical variables

**FIGURE 2:**
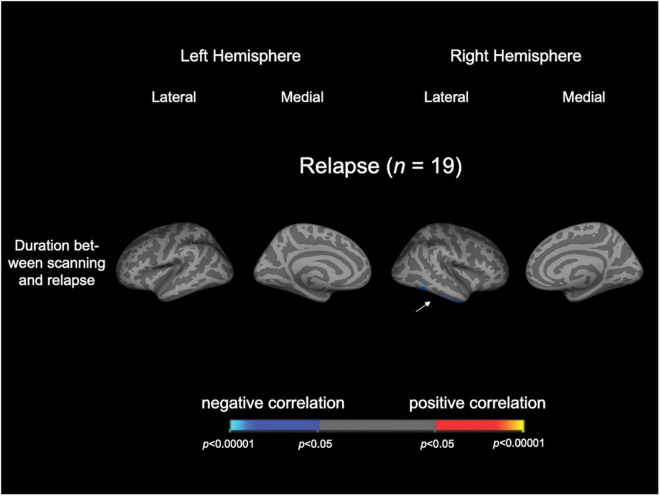
Cortical statistical maps displaying the significant correlation of local gyrification index with clinical variables in patients with schizophrenia. The maps are shown for the right and left hemispheres in lateral and medial views. An arrowhead in the figure indicates the location of clusters associated with time to relapse. Horizontal bar shows *p*-values (*p* < 0.05, corrected).

In the Relapse group (*n* = 19), the duration between scanning and first relapse was negatively correlated with LGI in the right inferior temporal gyrus and right fusiform gyrus; on the other hand, the number of relapses was not correlated with LGI in any region (Figure 2 and Table 3). When we also included medication dose and duration as controlling factors, significant clusters were similar to the original correlational results (Supplementary Figure 2).

**TABLE 3:** Clusters with significant correlation of local gyrification index with clinical variables.

Cluster no.	Cluster size (mm_2_)	Cluster-wise *p (corrected)*	MNI coordinates			Annotation
			*x*	*y*	*z*	
**Local gyrification index × duration between scanning and relapse**						
1	1125.22	0.0009	44.4	–10.7	–33.0	Right inferior temporal gyrus, fusiform gyrus

### Single regression analysis

The results of analysis of variance for model fit were not significant. The LGI values of any clusters were not associated with DUP (Supplementary Table 1).

## Discussion

To the best of our knowledge, this is the first MRI study to demonstrate a significant relationship between the brain gyrification pattern in first-episode schizophrenia and subsequent relapse. The Relapse group exhibited a significantly higher LGI in the bilateral parietal and left occipital areas compared with the Non-relapse group, suggesting that the two schizophrenia subgroups had distinct neurodevelopmental anomalies. In the Relapse group, the higher LGI in the right inferior temporal cortex at baseline was significantly associated with earlier relapse during clinical follow-up. This suggests that the degree of LGI changes in the temporo-parieto-occipital regions may be related to relapse susceptibility in first-episode schizophrenia.

In the present study, we demonstrated that the parietal and occipital LGIs were higher in the Relapse group, possibly reflecting the neurobiological heterogeneity of schizophrenia (38). Cortical distribution of LGI increase in the present Relapse group was similar to that observed in previous studies of schizophrenia (25, 39–42) and clinical high-risk individuals with later transition to psychotic disorders (22), suggesting that the underpinnings of susceptibility to relapses may be associated with the mechanisms of psychosis itself (43). Because increased LGI implicates altered structural connectivity (44–46), our results are partly consistent with previous findings that schizophrenia patients with treatment resistance, which could be closely related to the likelihood of relapse (47, 48), exhibited white matter integrity deficits in multiple tracts connecting widespread cortical areas including parietal and occipital areas (49–51). In contrast, our gyrification finding was incongruent with the previous study by Palaniyappan et al. (24) that reported a reduced fronto-temporal LGI in schizophrenia patients with poor treatment response. The reason for this discrepancy was unspecified, but different sample characteristics between the studies may be partly relevant as Palaniyappan et al. (24) examined older (mean = 28.1 years, *SD* = 7.9) and more heterogeneous (both affective and non-affective psychosis) patients compared to the present study. Of note, dysconnectivity of the default mode network, which includes precuneus and lateral parietal cortices as nodes, has been reported to imply disturbed self-referential and integrative processes (52, 53). Therefore, difficulties in controlling positive symptoms, including ego disorder, may lead to treatment resistance (54, 55). Future novel system-level analyses, such as connectome analyses (56), will be necessary to clarify the pathological role of parieto-occipital gyrification changes in first-episode schizophrenia.

The present study indicated that increased LGI in the inferior part of right temporal lobe may contribute to triggering an earlier psychotic relapse. A previous study reported that higher dopamine synthesis capacity was related to a shorter time to relapse in first-episode psychosis (7), but no brain morphological features are known to predict the time to relapse in schizophrenia. On the other hand, previous studies have demonstrated that the gyrification pattern of the right temporal cortex, including inferior temporal and fusiform gyri in first-episode schizophrenia, may contribute to susceptibility of positive psychotic symptomatology (25, 57). In particular, abnormal connectivity of the inferior longitudinal fasciculus, which is responsible for face recognition (58) and semantic processing (59), may be involved in vulnerability to delusion formation (60). Thus, brain neurodevelopmental pathologies involved in the formation of psychotic symptoms may be involved in early relapse after the first episode of schizophrenia.

Identifying neurobiological predictors of later psychotic relapses would pave the way for precision medicine that could be useful for one third of schizophrenia patients that are not benefiting from the current treatment strategies (4, 6). Based on stratification using biomarkers of relapse, patients at lower risk of relapse can be treated with no or low-dose antipsychotic treatment, whereas early initiation of clozapine may be considered for patients at higher risk of relapse (4, 61, 62). While a previous positron emission tomography study by Kim et al. (7) successfully reported that dynamic changes in striatal dopamine synthesis capacity could distinguish a Relapse group from a Non-relapse group as a state-specific marker, our study suggested the role of trait-specific brain characteristics (gyrification pattern) as a marker of relapse susceptibility. Our results may have future clinical implications, as the creation of a highly accurate prognostic discriminator using multimodal measures, especially including trait-specific markers, may lead to better outcomes by providing optimal treatment for each patient (12, 63).

This study had several limitations. First, most schizophrenia patients included in the present study were taking antipsychotics at scanning, which may have an effect on brain structure (64). However, as shown in the supplementary analyses (Supplementary Figures 1, 2), our finding of deviated gyrification patterns and their relation to relapse in schizophrenia could not be explained only by effect of medication. Second, while the clinical follow-up period in this study (3 years) was longer than conventional treatment response studies (3–24 weeks) (65–67), further long-term follow-up would be required to establish the role of brain gyrification as a predictor of clinical course of schizophrenia. Third, the relapse rate in our sample (36.5%) was lower than that in previous studies (e.g., 63.1% within 3 years) (1) possibly due to our successful clinical management, suggesting that relapsed patients in our cohort may not represent the full spectrum of relapsed patients. Fourth, the sample size of our cohort (especially relapsed patients) was relatively small and the statistical analysis for 800 ROIs required a high number of tests. The reproducibility and generality of the findings should be tested in more larger cohorts with appropriate multiple comparison correction for future clinical application. In this study, we failed to detect significant relationship between DUP, which was somewhat longer in relapsed than in non-relapsed patients (Table 1), and brain gyrification. Because longer DUP due to insidious illness onset, which could be related to prominent neurodevelopmental pathology (68), significantly affects later clinical course of schizophrenia (1, 8), future studies in larger cohorts are needed also for clarifying whether DUP is associated with relapse and/or brain morphology. Fifth, we did not control for some potential confounding factors, such as smoke, coffee/tea intake, and time of day for MRI scanning (69). Lastly, the definition of relapse has been inconsistent among previous studies (70); however, the definition in this study has been commonly used in other biological studies (71, 72). In addition, we have not objectively defined the clinical stability after their first episode as the starting point for a follow-up assessment of relapse. Furthermore, the causal relationship between relapse and other biological/clinical factors is somewhat complicated (12). Therefore, the role of potential influential factors, such as the treatment during follow-up, adherence to medication, and several environmental factors, on the present findings should be further examined.

## Conclusion

In conclusion, this whole-brain LGI study of first-episode schizophrenia elucidated that patients who later relapsed and those who did not relapse had distinct gyrification patterns already at illness onset, and that the anomalous gyrification patterns in relapsed patients were related to the time to first relapse. Increased LGI in the temporo-parieto-occipital regions in first-episode schizophrenia patients may be a potential susceptibility marker for subsequent relapses.

## Data availability statement

The datasets utilized for this article are not available immediately because we do not have permission to share them. Requests to access the datasets should be directed to DS, ds179@med.u-toyama.ac.jp.

## Ethics statement

The studies involving human participants were reviewed and approved by the Committee on the Medical Ethics of Toyama University. Written informed consent was received from all study participants. If the participants were under the age of 20, their parent or guardian also provided written consent.

## Author contributions

DS, YT, TT, and MS conceived the present study and its methods. DS conducted statistical analyses and wrote the manuscript. DS, AF, and HK recruited the participants and were involved in clinical and diagnostic assessment. DS analyzed MRI data. KN provided technical support for MRI and data processing. DS, AF, HK, and TT managed MRI and clinical data. TT and MS contributed to the writing and editing of the manuscript. All authors contributed to the article approved the final manuscript.

## Footnotes

1
https://surfer.nmr.mgh.harvard.edu

2
https://surfer.nmr.mgh.harvard.edu/fswiki/FreeSurferSupport

## References

[1] Robinson D Woerner MG Alvir JM Bilder R Goldman R Geisler S Predictors of relapse following response from a first episode of schizophrenia or schizoaffective disorder. *Arch Gene Psychiatr.* (1999) 56:241–7. 10.1001/archpsyc.56.3.241 10078501

[2] Almond S Knapp M Francois C Toumi M Brugha T. Relapse in schizophrenia: costs, clinical outcomes and quality of life. *Br J Psychiatry.* (2004) 184:346–51. 15056580 10.1192/bjp.184.4.346

[3] Takeuchi H Siu C Remington G Fervaha G Zipursky RB Foussias G Does relapse contribute to treatment resistance? Antipsychotic response in first- vs. second-episode schizophrenia. *Neuropsychopharmacology.* (2019) 44:1036–42. 10.1038/s41386-018-0278-3 30514883 PMC6462044

[4] Rubio JM Malhotra AK Kane JM. Towards a framework to develop neuroimaging biomarkers of relapse in schizophrenia. *Behav Brain Res.* (2021) 402:113099. 10.1016/j.bbr.2020.113099 33417996

[5] Leucht S Tardy M Komossa K Heres S Kissling W Davis JM. Maintenance treatment with antipsychotic drugs for schizophrenia. *Cochr Datab Syst Rev.* (2012) 2012:Cd008016. 10.1002/14651858.CD008016.pub2 22592725

[6] Correll CU Rubio JM Kane JM. What is the risk-benefit ratio of long-term antipsychotic treatment in people with schizophrenia? *World Psychiatry.* (2018) 17:149–60. 29856543 10.1002/wps.20516 PMC5980517

[7] Kim S Shin SH Santangelo B Veronese M Kang SK Lee JS Dopamine dysregulation in psychotic relapse after antipsychotic discontinuation: an [(18)F]DOPA and [(11)C]raclopride PET study in first-episode psychosis. *Mol Psychiatry.* (2021) 26:3476–88. 32929214 10.1038/s41380-020-00879-0

[8] Hui CL Honer WG Lee EH Chang WC Chan SK Chen ES Predicting first-episode psychosis patients who will never relapse over 10 years. *Psychol Med.* (2019) 49:2206–14. 10.1017/S0033291718003070 30375301

[9] Andreasen NC Nopoulos P Magnotta V Pierson R Ziebell S Ho BC. Progressive brain change in schizophrenia: a prospective longitudinal study of first-episode schizophrenia. *Biol Psychiatry.* (2011) 70:672–9. 21784414 10.1016/j.biopsych.2011.05.017 PMC3496792

[10] Tandon R Nasrallah HA Keshavan MS. Schizophrenia, “just the facts” 4. Clinical features and conceptualization. *Schizophr Res.* (2009) 110:1–23. 19328655 10.1016/j.schres.2009.03.005

[11] Agid O Arenovich T Sajeev G Zipursky RB Kapur S Foussias G An algorithm-based approach to first-episode schizophrenia: response rates over 3 prospective antipsychotic trials with a retrospective data analysis. *J Clin Psychiatry.* (2011) 72:1439–44. 10.4088/JCP.09m05785yel 21457676

[12] Remington G Foussias G Agid O Fervaha G Takeuchi H Hahn M. The neurobiology of relapse in schizophrenia. *Schizophr Res.* (2014) 152:381–90. 24206930 10.1016/j.schres.2013.10.009

[13] Demjaha A MacCabe JH Murray RM. How genes and environmental factors determine the different neurodevelopmental trajectories of schizophrenia and bipolar disorder. *Schizophrenia Bull.* (2012) 38:209–14. 10.1093/schbul/sbr100 21857009 PMC3283142

[14] Takahashi T Suzuki M. Brain morphologic changes in early stages of psychosis: implications for clinical application and early intervention. *Psychiatry Clin Neurosci.* (2018) 72:556–71. 10.1111/pcn.12670 29717522

[15] Armstrong E Schleicher A Omran H Curtis M Zilles K. The ontogeny of human gyrification. *Cereb Cortex.* (1995) 5:56–63. 7719130 10.1093/cercor/5.1.56

[16] Zilles K Palomero-Gallagher N Amunts K. Development of cortical folding during evolution and ontogeny. *Trends Neurosci.* (2013) 36:275–84. 23415112 10.1016/j.tins.2013.01.006

[17] Haukvik UK Schaer M Nesvåg R McNeil T Hartberg CB Jönsson EG Cortical folding in Broca’s area relates to obstetric complications in schizophrenia patients and healthy controls. *Psychol Med.* (2012) 42:1329–37. 10.1017/S0033291711002315 22029970

[18] Smith GN Thornton AE Lang DJ MacEwan GW Kopala LC Su W Cortical morphology and early adverse birth events in men with first-episode psychosis. *Psychol Med.* (2015) 45:1825–37. 10.1017/S003329171400292X 25499574

[19] Zhang Y Inder TE Neil JJ Dierker DL Alexopoulos D Anderson PJ Cortical structural abnormalities in very preterm children at 7 years of age. *Neuroimage.* (2015) 109:469–79. 25614973 10.1016/j.neuroimage.2015.01.005 PMC4340728

[20] Matsuda Y Ohi K. Cortical gyrification in schizophrenia: current perspectives. *Neuropsychiatr Dis Treat.* (2018) 14:1861–9. 30050300 10.2147/NDT.S145273 PMC6055839

[21] Sasabayashi D Takahashi T Takayanagi Y Suzuki M. Anomalous brain gyrification patterns in major psychiatric disorders: a systematic review and transdiagnostic integration. *Transl Psychiatry.* (2021) 11:176. 10.1038/s41398-021-01297-8 33731700 PMC7969935

[22] Sasabayashi D Takayanagi Y Takahashi T Koike S Yamasue H Katagiri N Increased occipital gyrification and development of psychotic disorders in individuals with an at-risk mental state: a multicenter study. *Biol Psychiatry.* (2017) 82:737–45. 10.1016/j.biopsych.2017.05.018 28709499

[23] Das T Borgwardt S Hauke DJ Harrisberger F Lang UE Riecher-Rössler A Disorganized gyrification network properties during the transition to psychosis. *JAMA Psychiatry.* (2018) 75:613–22. 10.1001/jamapsychiatry.2018.0391 29710118 PMC6137528

[24] Palaniyappan L Marques TR Taylor H Handley R Mondelli V Bonaccorso S Cortical folding defects as markers of poor treatment response in first-episode psychosis. *JAMA Psychiatry.* (2013) 70:1031–40. 10.1001/jamapsychiatry.2013.203 23945954 PMC7617342

[25] Sasabayashi D Takayanagi Y Nishiyama S Takahashi T Furuichi A Kido M Increased frontal gyrification negatively correlates with executive function in patients with first-episode schizophrenia. *Cereb Cortex.* (2017) 27:2686–94. 10.1093/cercor/bhw101 27095825

[26] Andreasen NC Flaum M Arndt S. The comprehensive assessment of symptoms and history (CASH). An instrument for assessing diagnosis and psychopathology. *Arch Gene Psychiatry.* (1992) 49:615–23. 10.1001/archpsyc.1992.01820080023004 1637251

[27] World Health Organization. *The ICD-10 Classification of Mental and Behavioural Disorders: Diagnostic Criteria for Research.* Geneva: World Health Organization (1993).

[28] Breitborde NJ Srihari VH Woods SW. Review of the operational definition for first-episode psychosis. *Early Intervent Psychiatry.* (2009) 3:259–65. 10.1111/j.1751-7893.2009.00148.x PMC4451818 22642728

[29] Andreasen NC. *Scale for the Assessment of Negative Symptoms (SANS).* Iowa, IA: University of Iowa Press (1983).

[30] Andreasen NC. *Scale for the Assessment of Positive Symptoms (SAPS).* Iowa, IA: University of Iowa (1984).

[31] Toru M. *Psychotropic Manual.* 3rd ed. Tokyo: Igaku-Shoin (2008).

[32] Hoff AL Sakuma M Razi K Heydebrand G Csernansky JG DeLisi LE. Lack of association between duration of untreated illness and severity of cognitive and structural brain deficits at the first episode of schizophrenia. *Am. J. Psychiatry.* (2000) 157:1824–8. 10.1176/appi.ajp.157.11.1824 11058480

[33] Csernansky JG Mahmoud R Brenner R. A comparison of risperidone and haloperidol for the prevention of relapse in patients with schizophrenia. *N Engl J Med.* (2002) 346:16–22. 11777998 10.1056/NEJMoa002028

[34] Fischl B. FreeSurfer. *Neuroimage.* (2012) 62:774–81. 22248573 10.1016/j.neuroimage.2012.01.021 PMC3685476

[35] Zilles K Armstrong E Schleicher A Kretschmann HJ. The human pattern of gyrification in the cerebral cortex. *Anat Embryol.* (1988) 179:173–9. 10.1007/BF00304699 3232854

[36] Schaer M Cuadra MB Tamarit L Lazeyras F Eliez S Thiran JP. A surface-based approach to quantify local cortical gyrification. *IEEE Transact Med Imaging.* (2008) 27:161–70. 10.1109/TMI.2007.903576 18334438

[37] Hagler DJ Jr. Saygin AP Sereno MI. Smoothing and cluster thresholding for cortical surface-based group analysis of fMRI data. *Neuroimage.* (2006) 33:1093–103. 17011792 10.1016/j.neuroimage.2006.07.036 PMC1785301

[38] Takayanagi Y Sasabayashi D Takahashi T Komori Y Furuichi A Kido M Altered brain gyrification in deficit and non-deficit schizophrenia. *Psychol Med.* (2019) 49:573–80. 10.1017/S0033291718001228 29739476

[39] Schultz CC Koch K Wagner G Roebel M Nenadic I Gaser C Increased parahippocampal and lingual gyrification in first-episode schizophrenia. *Schizophr Res.* (2010) 123:137–44. 20850277 10.1016/j.schres.2010.08.033

[40] Tepest R Schwarzbach CJ Krug B Klosterkötter J Ruhrmann S Vogeley K. Morphometry of structural disconnectivity indicators in subjects at risk and in age-matched patients with schizophrenia. *Eur Arch Psychiatry Clin Neurosci.* (2013) 263:15–24. 10.1007/s00406-012-0343-6 22821623

[41] Sasabayashi D Takayanagi Y Takahashi T Nemoto K Furuichi A Kido M Increased brain gyrification in the schizophrenia spectrum. *Psychiatry Clin Neurosci.* (2020) 74:70–6. 31596011 10.1111/pcn.12939

[42] Schultz CC Wagner G Koch K Gaser C Roebel M Schachtzabel C The visual cortex in schizophrenia: alterations of gyrification rather than cortical thickness–a combined cortical shape analysis. *Brain Struct Funct.* (2013) 218:51–8. 10.1007/s00429-011-0374-1 22200883

[43] Kelley ME Yao JK van Kammen DP. Plasma catecholamine metabolites as markers for psychosis and antipsychotic response in schizophrenia. *Neuropsychopharmacology.* (1999) 20:603–11. 10327429 10.1016/S0893-133X(98)00094-3

[44] Schaer M Ottet MC Scariati E Dukes D Franchini M Eliez S Decreased frontal gyrification correlates with altered connectivity in children with autism. *Front Hum Neurosci.* (2013) 7:750. 10.3389/fnhum.2013.00750 24265612 PMC3820980

[45] Ecker C Andrews D Dell’Acqua F Daly E Murphy C Catani M Relationship between cortical gyrification, white matter connectivity, and autism spectrum disorder. *Cereb Cortex.* (2016) 26:3297–309. 27130663 10.1093/cercor/bhw098 PMC4898679

[46] Schultz CC Wagner G Schachtzabel C Reichenbach JR Schlösser RG Sauer H Increased white matter radial diffusivity is associated with prefrontal cortical folding deficits in schizophrenia. *Psychiatry Res Neuroimag.* (2017) 261:91–5. 10.1016/j.pscychresns.2017.01.011 28171781

[47] Emsley R Nuamah I Hough D Gopal S. Treatment response after relapse in a placebo-controlled maintenance trial in schizophrenia. *Schizophr Res.* (2012) 138:29–34. 10.1016/j.schres.2012.02.030 22446143

[48] Emsley R Oosthuizen P Koen L Niehaus D Martinez L. Comparison of treatment response in second-episode versus first-episode schizophrenia. *J Clin Psychopharmacol.* (2013) 33:80–3. 10.1097/JCP.0b013e31827bfcc1 23277247

[49] Reis Marques T Taylor H Chaddock C Dell’acqua F Handley R Reinders AA White matter integrity as a predictor of response to treatment in first episode psychosis. *Brain J Neurol.* (2014) 137:172–82. 10.1093/brain/awt310 PMC3891445 24253201

[50] McNabb CB Kydd R Sundram F Soosay I Russell BR. Differences in white matter connectivity between treatment-resistant and treatment-responsive subtypes of schizophrenia. *Psychiatry Res Neuroimaging.* (2018) 282:47–54. 10.1016/j.pscychresns.2018.11.002 30412902

[51] Ochi R Noda Y Tsuchimoto S Tarumi R Honda S Matsushita K White matter microstructural organizations in patients with severe treatment-resistant schizophrenia: a diffusion tensor imaging study. *Prog Neuro Psychopharmacol Biol Psychiatry.* (2020) 100:109871. 10.1016/j.pnpbp.2020.109871 31962187

[52] Raichle ME MacLeod AM Snyder AZ Powers WJ Gusnard DA Shulman GL. A default mode of brain function. *Proc Natl Acad Sci USA.* (2001) 98:676–82. 11209064 10.1073/pnas.98.2.676 PMC14647

[53] Greicius MD Krasnow B Reiss AL Menon V. Functional connectivity in the resting brain: a network analysis of the default mode hypothesis. *Proc Natl Acad Sci USA.* (2003) 100:253–8. 10.1073/pnas.0135058100 12506194 PMC140943

[54] Lee WH Doucet GE Leibu E Frangou S. Resting-state network connectivity and metastability predict clinical symptoms in schizophrenia. *Schizophr Res.* (2018) 201:208–16. 10.1016/j.schres.2018.04.029 29709491 PMC6317903

[55] Wada M Nakajima S Tarumi R Masuda F Miyazaki T Tsugawa S Resting-State isolated effective connectivity of the cingulate cortex as a neurophysiological biomarker in patients with severe treatment-resistant schizophrenia. *J Pers Med.* (2020) 10:89. 10.3390/jpm10030089 32823914 PMC7564631

[56] Liu Z Palaniyappan L Wu X Zhang K Du J Zhao Q Resolving heterogeneity in schizophrenia through a novel systems approach to brain structure: individualized structural covariance network analysis. *Mol Psychiatry.* (2021) 26:7719–31. 34316005 10.1038/s41380-021-01229-4

[57] Takahashi T Sasabayashi D Takayanagi Y Furuichi A Kido M Nakamura M Altered Heschl’s gyrus duplication pattern in first-episode schizophrenia. *Schizophr Res.* (2021) 237:174–81. 10.1016/j.schres.2021.09.011 34536751

[58] Tavor I Yablonski M Mezer A Rom S Assaf Y Yovel G. Separate parts of occipito-temporal white matter fibers are associated with recognition of faces and places. *Neuroimage.* (2014) 86:123–30. 10.1016/j.neuroimage.2013.07.085 23933304

[59] Saur D Schelter B Schnell S Kratochvil D Küpper H Kellmeyer P Combining functional and anatomical connectivity reveals brain networks for auditory language comprehension. *Neuroimage.* (2010) 49:3187–97. 10.1016/j.neuroimage.2009.11.009 19913624

[60] Pankow A Knobel A Voss M Heinz A. Neurobiological correlates of delusion: beyond the salience attribution hypothesis. *Neuropsychobiology.* (2012) 66:33–43. 10.1159/000337132 22797275

[61] Okhuijsen-Pfeifer C Huijsman EAH Hasan A Sommer IEC Leucht S Kahn RS Clozapine as a first- or second-line treatment in schizophrenia: a systematic review and meta-analysis. *Acta Psychiatr Scand.* (2018) 138:281–8. 30218445 10.1111/acps.12954 PMC6175356

[62] Shah P Iwata Y Plitman E Brown EE Caravaggio F Kim J The impact of delay in clozapine initiation on treatment outcomes in patients with treatment-resistant schizophrenia: a systematic review. *Psychiatry Res.* (2018) 268:114–22. 10.1016/j.psychres.2018.06.070 30015109

[63] Wada M Noda Y Iwata Y Tsugawa S Yoshida K Tani H Dopaminergic dysfunction and excitory/inhibitory imbalance in treatment-resistant schizophrenia and novel neuromodulatory treatment. *Mol Psychiatry.* (2022) 27:2950–67. 10.1038/s41380-022-01572-0 35444257

[64] Tomelleri L Jogia J Perlini C Bellani M Ferro A Rambaldelli G Brain structural changes associated with chronicity and antipsychotic treatment in schizophrenia. *Eur Neuropsychopharmacol.* (2009) 19:835–40. 19717283 10.1016/j.euroneuro.2009.07.007

[65] Arango C Breier A McMahon R Carpenter WT Jr. Buchanan RW. The relationship of clozapine and haloperidol treatment response to prefrontal, hippocampal, and caudate brain volumes. *Am J Psychiatry.* (2003) 160:1421–7. 10.1176/appi.ajp.160.8.1421 12900303

[66] Molina V Martín C Ballesteros A de Herrera AG Hernández-Tamames JA. Optimized voxel brain morphometry: association between brain volumes and the response to atypical antipsychotics. *Eur Arch Psychiatry Clin Neurosci.* (2011) 261:407–16. 10.1007/s00406-010-0182-2 21191610

[67] Zugman A Gadelha A Assunção I Sato J Ota VK Rocha DL Reduced dorso-lateral prefrontal cortex in treatment resistant schizophrenia. *Schizophr Res.* (2013) 148:81–6. 10.1016/j.schres.2013.05.002 23721966

[68] Chen EY Hui CL Chan RC Dunn EL Miao MY. A 3-year prospective study of neurological soft signs in first-episode schizophrenia. *Schizophr Res.* (2005) 75:45–54. 10.1016/j.schres.2004.09.002 15820323

[69] Schneider CE White T Hass J Geisler D Wallace SR. Smoking status as a potential confounder in the study of brain structure in schizophrenia. *J Psychiatric Res.* (2014) 50:84–91. 10.1016/j.jpsychires.2013.12.004 24373929 PMC4047795

[70] Moncrieff J Crellin NE Long MA Cooper RE Stockmann T. Definitions of relapse in trials comparing antipsychotic maintenance with discontinuation or reduction for schizophrenia spectrum disorders: a systematic review. *Schizophr Res.* (2020) 225:47–54. 31604607 10.1016/j.schres.2019.08.035

[71] Pillai A Schooler NR Peter D Looney SW Goff DC Kopelowicz A Predicting relapse in schizophrenia: is BDNF a plausible biological marker? *Schizophr Res.* (2018) 193:263–8. 10.1016/j.schres.2017.06.059 28734907

[72] Mi L Wang L Li X She S Li H Huang H Reduction of phonetic mismatch negativity may depict illness course and predict functional outcomes in schizophrenia. *J Psychiatr Res.* (2021) 137:290–7. 10.1016/j.jpsychires.2021.02.065 33735719

