# Supplementary material for: Increased brain gyrification and subsequent relapse in patients with first-episode schizophrenia
Source: Front Psychiatry. 2022 Aug 10;13:937605. doi: 10.3389/fpsyt.2022.937605 (PMC9406142; doi:10.3389/fpsyt.2022.937605)
Supplement: Supplementary file 1 [file Data_Sheet_1.docx]

**Supplementary Material**

**Supplementary Figure S1.** Cortical statistical maps displaying the comparison of local gyrification index between relapsed and non-relapsed patients with schizophrenia (also controlling for medication dose and duration).

**Supplementary Figure S2.** Cortical statistical maps displaying the significant correlation of local gyrification index with clinical variables in patients with schizophrenia (also controlling for medication dose and duration).

**Supplementary Table S1.** Single linear regression analyses for average LGI values in three clusters where we found significant differences between Relapse and Non-Relapse groups.

**Supplementary Figure S1. Cortical statistical maps displaying the comparison of local gyrification index between relapsed and non-relapsed patients with schizophrenia (also controlling for medication dose and duration).**

Cortical statistical maps displaying increased local gyrification index in first-episode schizophrenia patients who experienced relapse (Relapse, *n* = 19) compared with those who remained relapse-free during the follow-up (Non-relapse, *n* = 33) using different controlling factors (duration of medication and medication dose in addition to age and sex). In antipsychotic-free/naïve patients, daily dosage or duration of antipsychotic medication were set to 0. Horizontal bar shows *p* values (*p* < 0.05, corrected).

**Supplementary Figure S2. Cortical statistical maps displaying the significant correlation of local gyrification index with clinical variables in patients with schizophrenia (also controlling for medication dose and duration).**

Cortical statistical maps displaying a negative correlation between local gyrification index and duration between scanning and first relapse in relapsed patients with schizophrenia (Relapse, *n* = 19). Age, sex, duration of medication, and medication dose were used as controlling factors. In antipsychotic-free/naïve patients, daily dosage or duration of antipsychotic medication were set to 0. Arrowhead in the figure indicates the location of clusters associated with time to relapse. Horizontal bar shows *p* values (*p* < 0.05, corrected).
